# Supervised Rehabilitation May Lead to Better Outcome than Home-Based Rehabilitation Up to 1 Year after Anterior Cruciate Ligament Reconstruction

**DOI:** 10.3390/medicina57010019

**Published:** 2020-12-28

**Authors:** Hye Chang Rhim, Jin Hyuck Lee, Seo Jun Lee, Jin Sung Jeon, Geun Kim, Kwang Yeol Lee, Ki-Mo Jang

**Affiliations:** 1 Department of Orthopaedic Surgery, Anam Hospital, Korea University College of Medicine, Seoul 02841, Korea; hr233@cornell.edu (H.C.R.); lsjohn236@gmail.com (S.J.L.); ixxi89js@gmail.com (J.S.J.); haru_ever93@naver.com (G.K.); lkr0817@naver.com (K.Y.L.); 2 Department of Sports Medical Center, Anam Hospital, Korea University College of Medicine, Seoul 02841, Korea; gnkfccc@hanmail.net

**Keywords:** anterior cruciate ligament, anterior cruciate ligament reconstruction, supervised rehabilitation, home-based rehabilitation, muscle strength, neuromuscular control

## Abstract

*Background and objectives:* Previous studies consistently found no significant difference between supervised and home-based rehabilitation after anterior cruciate ligament reconstruction (ACLR). However, the function of the nonoperative knee, hamstring strength at deep flexion, and neuromuscular control have been overlooked. This prospective observational study was performed to investigate the outcomes after ACLR in operative and nonoperative knees between supervised and home-based rehabilitations. *Materials and Methods:* After surgery, instructional videos demonstrating the rehabilitation process and exercises were provided for the home-based rehabilitation group. The supervised rehabilitation group visited our sports medicine center and physical therapists followed up all patients during the entire duration of the study. Isokinetic muscle strength and neuromuscular control (acceleration time (AT) and overall stability index (OSI)) of both operative and nonoperative knees, as well as patient-reported knee function (Lysholm score), were measured and compared between the two groups 6 months and 1 year postoperatively. *Results:* The supervised rehabilitation group showed higher muscle strength of hamstring and quadriceps in nonoperative knees at 6 months (hamstring, *p* = 0.033; quadriceps, *p* = 0.045) and higher hamstring strength in operative and nonoperative knees at 1 year (operative knees, *p* = 0.035; nonoperative knees, *p* = 0.010) than the home-based rehabilitation group. At 6 months and 1 year, OSIs in operative and nonoperative knees were significantly better in the supervised rehabilitation group than in the home-based rehabilitation group (operative knees, *p* < 0.001, *p* < 0.001; nonoperative knees, *p* < 0.001, *p* < 0.001, at 6 months and 1 year, respectively). At 1 year, the supervised rehabilitation group also demonstrated faster AT of the hamstrings (operative knees, *p* = 0.016; nonoperative knees, *p* = 0.036). Lysholm scores gradually improved in both groups over 1 year; however, the supervised rehabilitation group showed higher scores at 1 year (87.3 ± 5.8 vs. 75.6 ± 15.1, *p* = 0.016). *Conclusions:* This study demonstrated that supervised rehabilitation may offer additional benefits in improving muscle strength, neuromuscular control, and patient-reported knee function compared with home-based rehabilitation up to 1 year after ACLR.

## 1. Introduction

Anterior cruciate ligament (ACL) injury is common in sports involving cutting, jumping, and pivoting movements, and often requires ACL reconstruction (ACLR) followed by postoperative rehabilitation [1,2,3]. The purpose of ACLR and rehabilitation is to restore the stability and functional capacity of the ACL-deficient knee, thereby facilitating a return to the preinjury level of activity or sports participation [4]. Despite the high reported success rate of ACLR (>90%) [5,6,7], a retrospective analysis of 948 patients showed that only 69% of the patients were classified as normal or nearly normal according to the International Knee Documentation Committee (IKDC) evaluation system [8]. In addition, a previous systematic review showed that only 63% of the patients returned to their preinjury level of sports participation and 44% returned to competitive sports following ACLR [4].

Owing to the importance of postoperative rehabilitation following ACLR, previous studies investigated whether rehabilitation supervised by physical therapists would yield a superior outcome compared with home-based rehabilitation [7,9,10,11,12,13,14,15]. These studies consistently found no significant difference between supervised rehabilitation programs and home-based exercise programs. The outcome measures in the previous studies have included range of motion (ROM), muscle strength, hopping test results, Lysholm score, Tegner activity scale, and IKDC score [7,9,10,11,12,13,14,15]. Although these outcomes are the main interest of orthopedic surgeons or rehabilitation specialists, the function of the nonoperative knee, hamstring muscle strength at deep flexion, and neuromuscular control are overlooked measures that need to be properly addressed in order to prevent re-injury or new injury to the contralateral ACL and facilitate return to sports [1,16,17].

The purpose of this study was to investigate and compare the outcomes, including neuromuscular control and muscle strength, of both operative and nonoperative knees between a group of patients undergoing supervised physical therapy and a group of patients undergoing unsupervised home-based rehabilitation up to postoperative 1 year following ACLR. Lysholm scores were also compared between these two groups. We hypothesized that although there may be no differences in the muscle strength of the operative knees and the Lysholm scores between the two groups, there would be a difference in the muscle strength of the nonoperative knees, hamstring strength at deep flexion, and neuromuscular control.

## 2. Materials and Methods

### 2.1. Study Design

This prospective observational study aimed to compare functional outcomes between two groups of patients, a group undergoing supervised rehabilitation and a group undergoing home-based rehabilitation, at 6 months and 12 months. The study was approved by the institutional review board of the hospital to which the corresponding author is affiliated (IRB No.2018AN0261).

### 2.2. Patient Enrollment

Participants who were scheduled for ACLR at our institution from August 2018 to May 2019 and who could attend follow-up visits at 2 weeks, 6 weeks, 3 months, 6 months, and 1 year were candidates for the study. Patients with previous knee surgeries, with concomitant neurological diseases, who could not understand Korean, and who had visual impairment were not eligible. Patients with previous knee surgeries and concomitant neurological diseases were excluded because these conditions may affect the baseline and postoperative quadriceps and hamstring strength and neuromuscular control. Additionally, because physical therapists and instructional videos communicated in the Korean language, patients who could not understand Korean or had visual impairment were excluded. Patients who were willing to participate in the supervised rehabilitation program visited our sports medicine center once a week for 3 months. If the patients were unable to attend the supervised program, instructional videos were provided. Informed consent was acquired from all individual participants included in the study.

### 2.3. Surgical Technique

All operations were performed using the same technique (anatomical single-bundle ACLR using autologous hamstring tendon graft) under a spinal or general anesthesia by a single knee arthroscopic specialist. After the patient was positioned supine, a tourniquet was applied on proximal thigh and the limb was supported by a leg holder with the knee joint at 90° of flexion. Standard knee arthroscopic examination was performed through anterolateral and anteromedial portals using a 30°, 4.0-mm arthroscope. After a full intra-articular examination, any concomitant pathology of the articular cartilage and meniscus that required attention was corrected before ACLR. An approximately 3-cm skin incision was made anteromedially to the tibial tuberosity to harvest the semitendinosus and gracilis tendons, which were prepared using a standard tendon stripper. The harvested tendons were prepared on a back table and the ends of the grafts were sutured to form a closed loop. After a central mid-patellar portal was made, the femoral tunnel was created using the ACL RetroConstruction System and FlipCutter drill (Arthrex, Naples, FL, USA) through anatomic outside-in retrograde-reaming technique [18,19]. Then, the tibial tunnel was made with use of an ACL tibial guide. Finally, the prepared tendon graft was fixed using a bioabsorbable interference screw with a post tie for the tibial side and a TightRope^®^ RT (Arthrex, Naples, FL, USA) for the femoral side.

### 2.4. Home-Based Rehabilitation Group

Instructional videos demonstrating the rehabilitation process and exercises were provided. These videos were divided into goals and instructions over five stages, as described by Hohmann et al. and Cavanaugh and Powers [7,20]. A total of five videos were viewed during hospitalization for ACLR (the patients were admitted the day before ACLR and discharged 2 or 3 days after ACLR) and during follow-up visits at 2 weeks, 6 weeks, 3 months, and 6 months. Patients were allowed to advance to the next stage only if the goals of the previous stage had been met, and the patients’ progress was checked by the physical therapists during the follow-up visits.

### 2.5. Supervised Rehabilitation Group

Patients who chose to attend the supervised rehabilitation program visited our sports medicine center once a week for 3 months. The same physical therapist followed up all patients during the entire duration of the study. After 3 months, the patients were followed up bi-weekly or at longer intervals at the discretion of the physical therapist. The same-stage rehabilitation protocol for the home-based group was used for the supervised rehabilitation group.

### 2.6. Rehabilitation Protocol

During the first two weeks, the goal was to control postoperative pain and swelling and to gain ROM up to 90°. Then, the patients aimed to gradually gain ROM up to 130° and attain normal gait by postoperative 6 weeks. After this period, the patients focused on restoring muscle strength and proprioception. All the patients were braced up to 3 months postoperatively. Once the patients achieved enough strength and proprioception by 3 months, they could work on improving functional performance. After postoperative 6 months, the patients were permitted for gradual participation in sports activities if they achieved >85% of quadriceps and hamstring strengths and ROM >130 degrees. However, the types, frequency, and intensity of sports were tailored to each patient according to his or her physical and psychological conditions assessed by the physical therapists and orthopedic surgeon. Specific details of the rehabilitation protocol were based on the orthopedic textbook, “Campbell’s Operative Orthopedics” [21].

### 2.7. Assessment of Quadriceps and Hamstring Strength

Isokinetic quadriceps and hamstring strength (concentric/concentric muscle contraction for extension/flexion) was measured using Biodex multi-joint system 4 (Biodex Medical Systems, Shirley, NY, USA). Each patient was seated on the device with the hips and knees flexed to 90° and the trunk perpendicular to the floor. A strap was used to restrict each thigh, and the lateral femoral condyle of the knee joint was aligned with the rotational axis of the isokinetic machine. Each patient then performed a set that consisted of five isokinetic knee flexions and extensions (range of motion, 100–0°) of both operative and nonoperative legs at 180°/s, with a rest time of 30 s between sets. Flexion and extension strengths were measured by peak torque normalized to body weight (peak torque/body weight, [Nm/kg] × 100), and deep flexion strength was evaluated as the peak torque/body weight at 100° of knee flexion, which is an important factor in assessing the recovery of hamstring muscle strength after hamstring-harvesting ACLR [17,22]. Flexor strength and extensor strength were considered the hamstring muscle strength and quadriceps muscle strength, respectively. The mean values of two trials were considered the maximal peak torque for the hamstring and quadriceps muscles.

### 2.8. Assessment of Neuromuscular Control

Previous studies showed that the acceleration time (AT) can be a meaningful index of muscle activation and neuromuscular readiness to produce maximal muscular contraction [23,24,25]. In this study, AT was defined as the muscle activation time for attaining a pre-set angular velocity, which was 180°/s, during maximal muscle contraction [1]. Using Biodexmulti-joint system 4 (Biodex Medical Systems, Shirley, NY, USA), the ATs (in milliseconds) of the hamstring and quadriceps were measured during flexion/extension. A slower AT indicated a delay in neuromuscular control [25]. The patients were seated in the same position as that used for measuring isokinetic muscle strength values. Before testing, the patients performed a 5-min warm up, and then performed five submaximal repetitions of flexion and extension motions at 180°/s, followed by 15 maximal contractions at 180°/s after 1-min rest period. Gravity correction for torque was acquired in a relaxed state at 30°/s knee extension and calculated using the Biodex advantage software.

As another measure of neuromuscular control, we also assessed dynamic postural stability using the Biodex Stability System (BSS; Biodex Medical Systems, Shirley, NY, USA). Because a torn ACL can lead to compromised postural control and reduced knee stability [26,27], the evaluation of dynamic balance may be a good method for assessing the function of the sensory motor system after an ACL injury [28,29]. The BSS can position the foot platform surface up to 20°/s tilt in any direction. The patients were asked to perform the dynamic single-leg test while the stability level of the foot platform changed from level 12 (most stable) to level 1 (most unstable). The patients stood barefoot on the platform and were instructed to stand with 90°/s flexion of the opposite knee, with the arms held at the pelvis. The nonoperative knee was tested first, followed by the other knee, with 10-s rest intervals. The patients performed two trials in 20 s to measure postural stability. The mean and standard deviation of the two trials were calculated by the system (overall stability index, OSI). A lower stability index was considered good postural stability [30,31].

### 2.9. Patient-Reported Knee Function

The Lysholm score was used to evaluate the patient-reported knee function (limp, need for support, locking, instability, pain, swelling, and impairment of stair climbing and squatting ability). The Lysholm score ranges from 0 to 100, and is a valid outcome measure for patients with ACL injuries [32].

### 2.10. Statistical Analysis

On the basis of previous studies on quadriceps muscle strength in patients with knee joint injuries [1,3], a difference in quadriceps strength of at least >10% was considered clinically significant for the comparison between the supervised rehabilitation and home-based rehabilitation groups. A priori power analysis was used to determine the sample size, with an alpha level of 0.05 and a power of 0.8. A pilot study of five knees in each group was performed for sample size estimation. Twelve patients were required to detect a significant difference in the operative knees between the two groups (Cohen’s d: 1.925). The present study recruited 13 patients each for the supervised rehabilitation group and home-based rehabilitation group. The power of this study was 0.851 for detecting a significant difference between the two groups.

Repeated measures analysis of variance was used to compare three time points (preoperative baseline, postoperative 6 months, and postoperative 1 year) for the hamstring-to-quadriceps (H-Q) ratio, quadriceps and hamstring strength, AT, and dynamic postural stability. If a significant difference was found, a Bonferroni post hoc test was used to determine the differences in all variables, between the time points and between the groups. The Shapiro-Wilk test was used to determine whether a continuous variable followed a normal distribution. Statistical analysis was performed using the SPSS software version 21.0 (SPSS Inc., Chicago, IL, USA), and a value of *p* < 0.05 was considered statistically significant.

## 3. Results

### 3.1. Baseline Characteristics

No significant differences were found in age, height, weight, or body mass index (BMI) between the two groups. The detailed demographic data are presented in Table 1.

### 3.2. Serial Change of Quadriceps and Hamstring Strength in Each Group

In both the supervised and home-based rehabilitation groups, the H-Q ratio did not significantly change from the preoperative period to postoperative 6 months and 1 year; however, the muscle strength of hamstring and quadriceps in operative knees gradually improved over 6 months and 1 year (supervised rehabilitation group: hamstring, 55.0 ± 37.9, 95.2 ± 21.4, and 113.3 ± 21.8 (*p* < 0.001), quadriceps, 114.9 ± 68.2, 178.5 ± 37.7, and 227.7 ± 41.8 (*p* < 0.001), at baseline, 6 months, and 1 year, respectively; home-based rehabilitation: hamstring, 54.8 ± 26.3, 89.7 ± 30.6, and 93.4 ± 23.7 (*p* < 0.001), quadriceps, 106.6 ± 53.8, 144.8 ± 64.8, and 179.9 ± 77.2 (*p* < 0.001), at baseline, 6 months, and 1 year, respectively; Table 2 and Table 3).

In the supervised rehabilitation group, the hamstring muscle strength at 100° of knee flexion gradually improved over 6 months and 1 year in both operative and nonoperative knees (operative knees: 39.7 ± 25.9, 84.4 ± 22.6, and 98.9 ± 20.1 (*p* < 0.001) at baseline, 6 months, and 1 year, respectively; nonoperative knees: 93.4 ± 24.7, 103.1 ± 18.6, and 115.7 ± 24.7 (*p* = 0.010), at baseline, 6 months, and 1 year, respectively; Table 2). However, in the home-based rehabilitation group, the hamstring muscle strength at 100° of knee flexion gradually improved over 6 months and 1 year only in operative knees (29.6 ± 13.6, 53.2 ± 27.4, and 70.4 ± 19.2, (*p* < 0.001), at baseline, 6 months, and 1 year, respectively; Table 3).

### 3.3. Comparison of Quadriceps and Hamstring Strength between the Two Groups

**Figure 1:**
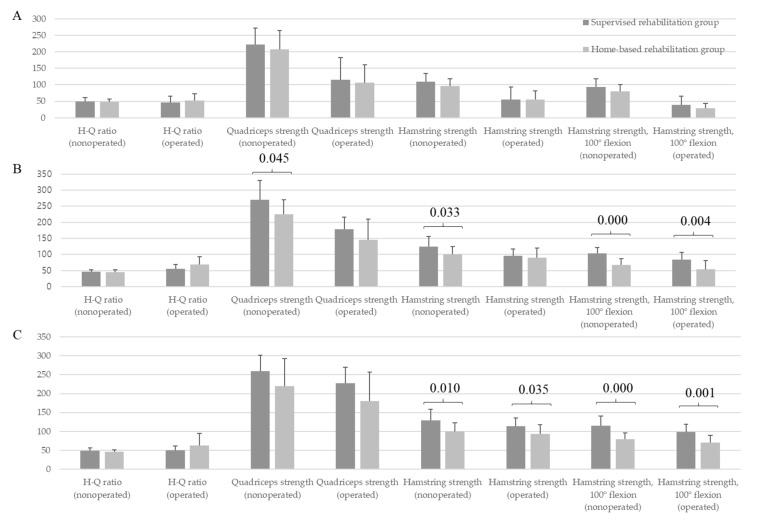
Comparison of muscle ratio and strength of quadriceps and hamstring between the supervised rehabilitation group and home-based rehabilitation group. (**A**) Preoperative baseline, (**B**) At postoperative 6 months, and (**C**) At postoperative 1 year.

The supervised rehabilitation group showed higher muscle strength of hamstring and quadriceps in nonoperative knees at 6 months (hamstring, 124.4 ± 30.9 vs. 100 ± 23.7 (*p* = 0.033); quadriceps, 269.8 ± 60.5 vs. 225.5 ± 44.8 (*p* = 0.045); Figure 1) and higher hamstring strength in operative and nonoperative knees at 1 year (operative knees, 113.3 ± 21.8 vs. 93.4 ± 23.7 (*p* = 0.035); nonoperative knees, 128.9 ± 29.9 vs. 100 ± 22.8 (*p* = 0.010); Figure 1) than the home-based rehabilitation group. However, no significant difference in the H-Q ratio of operative and nonoperative knees at the three time points between the two groups (*p* > 0.05, Figure 1).

The hamstring strength at deep flexion was significantly higher in both operative and nonoperative knees at 6 months and 1 year in the supervised rehabilitation group (operative knees, 84.4 ± 22.6 vs. 53.2 ± 27.4 (*p* = 0.004), 98.9 ± 20.1 vs. 70.4 ± 19.2 (*p* = 0.001); nonoperative knees, 103.1 ± 18.6 vs. 67.1 ± 19.4 (*p* < 0.001), 115.7 ± 24.7 vs. 78.8 ± 17.7 (*p* < 0.001), at 6 months and 1 year, respectively; Figure 1) than in the home-based rehabilitation group.

### 3.4. Serial Changes of Neuromuscular Control

In the supervised rehabilitation group, the AT of the hamstring and quadriceps and OSI in both operative and nonoperative knees gradually improved over 6 months and 1 year (operative knees: hamstring, 63.1 ± 11, 50.8 ± 10, and 32.3 ± 9 (*p* < 0.001), quadriceps, 47.7 ± 13, 40.8 ± 11, and 33.1 ± 10 (*p* = 0.002); nonoperative knees: hamstring, 55.4 ± 13, 49.2 ± 7, and 30.8 ± 11 (*p* < 0.001), quadriceps, 48.5 ± 12, 31.5 ± 6, and 26.2 ± 5 (*p* < 0.001), at baseline, 6 months, 1 year, respectively; Table 4). In the home-based rehabilitation group, the AT of the hamstring in both operative and nonoperative knees improved over 6 months and 1 year (operative knees: hamstring, 66.9 ± 25, 50 ± 24, and 47.7 ± 19 (*p* = 0.003); nonoperative knees: hamstring, 60.8 ± 24, 44.6 ± 11, and 43.8 ± 18 (*p* < 0.001), at baseline, 6 months, 1 year, respectively; Table 5); however, the AT of the quadriceps and the OSI did not significantly improve (*p* > 0.05, Table 5).

### 3.5. Comparison of the Neuromuscular Control between the Two Groups

**Figure 2:**
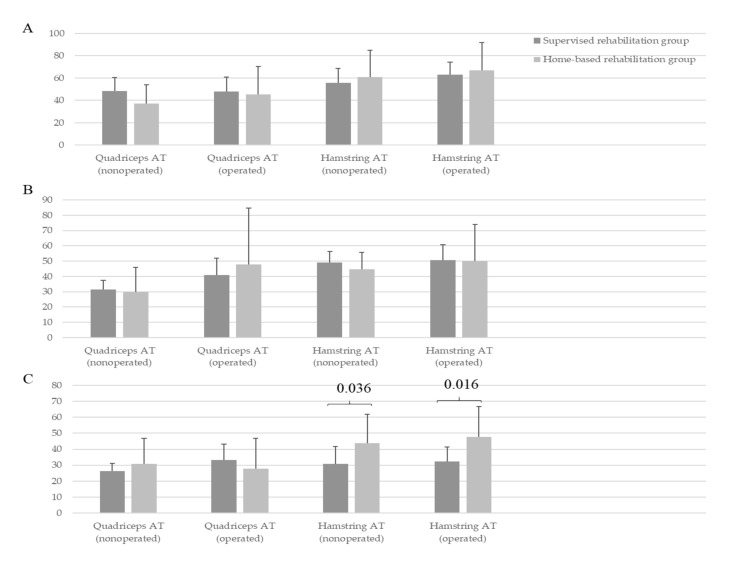
Comparison of acceleration time (AT) of quadriceps and hamstring in operative and nonoperative knees between the supervised rehabilitation group and home-based rehabilitation group. (**A**) Preoperative baseline, (**B**) At postoperative 6 months, and (**C**) At postoperative 1 year.

**Figure 3:**
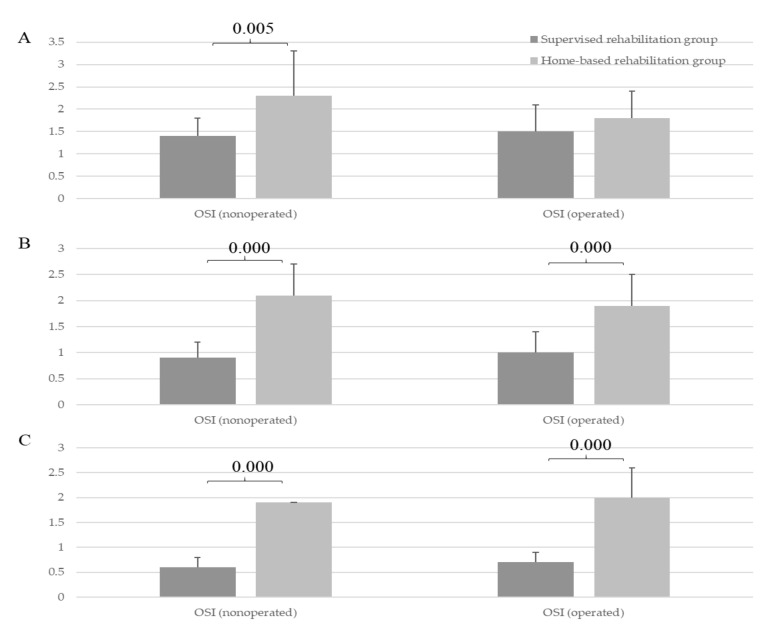
Comparison of dynamic postural stability (overall stability index, OSI) in operative and nonoperative knees between the supervised rehabilitation group and home-based rehabilitation group. (**A**) Preoperative baseline, (**B**) At postoperative 6 months, and (**C**) At postoperative 1 year.

No significant difference in the AT of the hamstring and quadriceps was found between the two groups at baseline and 6 months (*p* > 0.05, Figure 2); however, at 1 year, the supervised rehabilitation group showed better AT of the hamstring in both operative and nonoperative knees than the home-based rehabilitation group (operative knees, 32.3 ± 9 vs. 47.7 ± 19 (*p* = 0.016); nonoperative knees, 30.8 ± 11 vs. 43.8 ± 18 (*p* = 0.036); Figure 2). At baseline, the OSI of operative knees in the supervised rehabilitation group did not significantly differ from that in the home-based rehabilitation group (*p* > 0.05); however, the OSI of the nonoperative knees in the supervised rehabilitation group was significantly lower than that in the home-based group (1.4 ± 0.4 vs. 2.3 ± 1.0, *p* = 0.005; Figure 3). At 6 months and 1 year, the OSI of both operative and nonoperative knees in the supervised rehabilitation group was significantly lower than that in the home-based rehabilitation group (operative knees, 1.0 ± 0.4 vs. 1.9 ± 0.6 (*p* < 0.001), 0.7 ± 0.2 vs. 2.0 ± 0.6 (*p* < 0.001); nonoperative knees, 0.9 ± 0.3 vs. 2.1 ± 0.6 (*p* = 0.000), 0.6 ± 0.2 vs. 1.9 ± 0.9 (*p* < 0.001), at 6 months and 1 year, respectively; Figure 3).

### 3.6. Patient-Reported Knee Function

**Figure 4:**
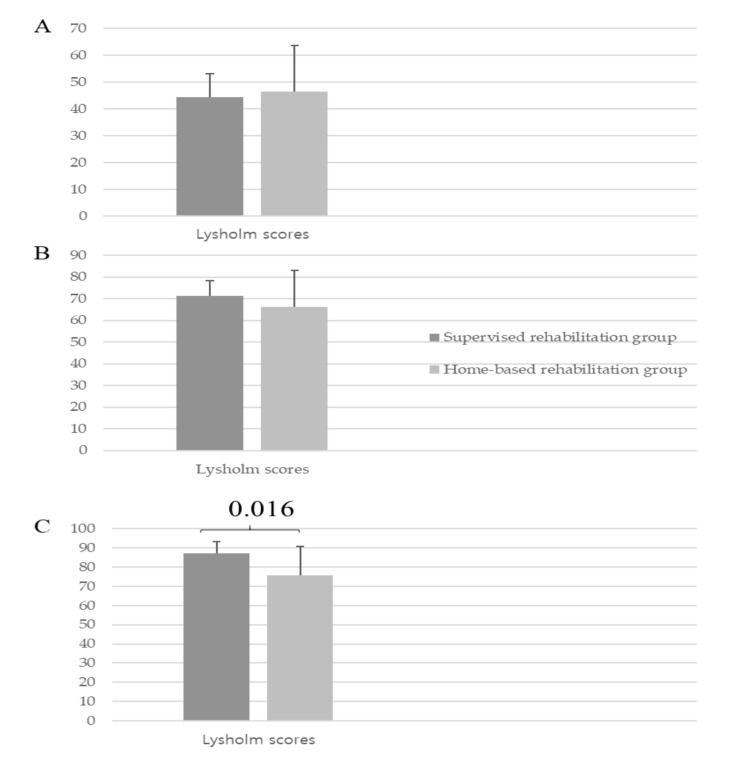
Comparison of the Lysholm scores between the supervised rehabilitation group and home-based rehabilitation group. (**A**) Preoperative baseline, (**B**) At postoperative 6 months, and (**C**) At postoperative 1 year.

In both groups, the Lysholm score gradually improved over 6 months and 1 year (supervised rehabilitation group, 44.3 ± 8.7, 71.3 ± 7.0, and 87.3 ± 5.8 (*p* < 0.001); home-based rehabilitation group, 46.3 ± 17.4, 66.3 ± 16.8, 75.6 ± 15.1 (*p* < 0.001), at baseline, 6 months, and 1 year, respectively; Table 6). However, at 1 year, the supervised rehabilitation group showed greater Lysholm score than the home-based rehabilitation group (87.3 ± 5.8 vs. 75.6 ± 15.1, *p* = 0.016; Figure 4).

## 4. Discussion

The most important finding of this study was that the supervised rehabilitation group demonstrated greater muscle strength, better neuromuscular control, and higher patient-reported knee function than the home-based rehabilitation group. Except for one study which found some benefits from the home-based rehabilitation in flexion and extension ROM at postoperative 3 months [13], previous studies have reported no significant difference in muscle strength, knee function, activity level, anterior tibial translation, ROM, hopping test results between home-based rehabilitation and supervised rehabilitation [7,9,10,12,14,15,33]. Grant el al. reported no significant difference in ACL quality-of-life questionnaire scores, knee range of motion, sagittal plane laxity, quadriceps and hamstring strength, and IKDC score between the two groups after following up the patients for 2–4 years [34]. The most recent systematic review concluded that reliable and compliant patients undergoing home-based rehabilitation can achieve the same results as those undergoing supervised rehabilitation [35]. However, our results suggest that supervised rehabilitation may have additional benefits over home-based rehabilitation.

### 4.1. Quadriceps and Hamstring Strength

One of the main outcome measures investigated in previous studies comparing supervised and home-based rehabilitation was quadriceps and hamstring strength [7,9,10,12,34]. However, evaluating the nonoperative knee is worthwhile, because a muscle strength deficit was observed in the uninjured limb after ACLR up to 40 months of follow-up [16,36,37]. Moreover, patients undergoing ACLR with medial hamstring tendon harvest were shown to have significant weakness of the hamstring muscle at high knee flexion angles [17]. In this respect, we evaluated the muscle strength of both operative and nonoperative knees and the hamstrings muscle strength at deep flexion angle. Unlike previous studies [7,9,10,12,34], we found greater deficits in isokinetic hamstring and quadriceps muscle strength at 6 months in nonoperative knees and in isokinetic hamstring muscle strength at 1 year in both operative and nonoperative knees in the home-based rehabilitation group. Moreover, the hamstring muscle strength at deep flexion in both operative and nonoperative knees was weaker at 6 months and 1 year in the home-based rehabilitation group.

### 4.2. Neuromuscular Control

Despite the importance of neuromuscular control in maintaining muscle activation and dynamic knee joint stability [1], there is still no universal test for objectively evaluating neuromuscular control after ACLR [38]. Unlike previous studies that used hopping tests [7,12,14], our study assessed AT and dynamic postural stability (OSI) for neuromuscular control. Although previous studies reported no significant difference in hopping test results between the two groups, our study demonstrated that the supervised rehabilitation group had better neuromuscular control at 6 months and 1 year. Moreover, similar to quadriceps and hamstring strength, we found a greater neuromuscular deficit in both nonoperative and operative knees in the home-based group. Given that previous studies have demonstrated that neuromuscular control could be impaired in nonoperative knees of patients with ACL injury [1,16,39,40,41], our findings suggest that supervised rehabilitation may prevent the loss of neuromuscular control not only in operative knees, but also in nonoperative knees.

### 4.3. Patient-Reported Knee Function

Previous studies comparing the Lysholm scores between the supervised and home-based rehabilitation groups reported no significant difference between the two groups at 6 months and 1 year [7,9,12]. Our results are partially consistent with those of previous studies in that no significant difference was observed at 6 months, but the supervised rehabilitation group showed higher Lysholm scores at 1 year than the home-based rehabilitation group. The difference was large enough to categorize the mean scores of these two groups into good (supervised rehabilitation group) and fair (home-based rehabilitation group) [42]. Given the reported minimal clinically important difference value of 10 for the Lysholm score [43], the difference between the two groups in the present study is significant and implies that supervised rehabilitation may offer additional benefits in improving patient-reported knee function.

### 4.4. Clinical Implications

To date, multiple studies have demonstrated contralateral muscle strength and neuromuscular control deficits after ACLR [1,16,36,37,39,40,41]. One of the possible explanations for this contralateral limb weakness is gamma motor neuron dysfunction [44,45,46]. The gamma loop runs from the mechanoreceptors in the ACL to the gamma motor neurons in order to produce maximal voluntary contraction. ACL injury causes impairment in the mechanoreceptors and a decrease in the afferent feedback from the ACL. This attenuates gamma motor neuron activation, which subsequently decreases the 1a afferents linked to the contralateral muscle via the supraspinal nervous system. As 1a feedback is required for voluntary contraction but becomes dysfunctional after ACL injury, the uninjured limb may also be affected by the injury in the other limb [16]. Despite such influence of ACL injury in both limbs, the results of our study suggest that patients in the home-based group might not have understood the importance of performing rehabilitation exercises for both limbs. Moreover, it is possible that patients in the home-based group performed rehabilitation exercises selectively, not as instructed. Conversely, patients in the supervised group performed the assigned exercises that were designed according to their progress and the specific number of sets for both limbs. This difference might explain why the supervised rehabilitation group showed higher muscle strength in both operative and nonoperative knees and better neuromuscular control. Given the increased risk of contralateral ACL injury with past ACL injury [47,48,49], the supervised rehabilitation group may have an advantage over the home-based group because supervision can better enhance muscle strength and neuromuscular control.

Another possible explanation for the difference between the two groups may be derived from the interaction with physical therapists. A previous systematic review identified that self-confidence, optimism, self-motivation, stress, social support, and athletic self-identity are predictors that affect ACLR outcomes [50]. Constant interaction with physical therapists during weekly visits in the first 3 months might have altered one of these psychological factors and improved the outcomes seen in the present study. However, further studies are needed to ascertain whether this interaction can elicit a meaningful difference in psychological states between the two groups.

### 4.5. Limitations

The present study had some limitations. First, as this was a nonrandomized, prospective observational study, a selection bias may have been present. Even though the demographic data showed no significant difference in sex, age, height, weight, and BMI between the two groups, patients with different psychological factors may have self-selected one rehabilitation program over the other. Second, preoperative OSI of nonoperative knees in the supervised rehabilitation group was significantly lower than that in the home-based group. Thus, the better neuromuscular control in the supervised rehabilitation group at 6 months and 1 year may be attributed to this baseline difference. Moreover, the home-based rehabilitation group had two more patients with concurrent meniscus tear, which might explain the worse neuromuscular control in this group [3]. Third, while the Tegner activity scale and IKDC score are also validated and reliable tools to measure functional outcomes following ACLR [32,42,51], the current study only used the Lysholm score. Fourth, this study enrolled a relatively small number of patients in each group. However, we performed a power analysis to determine the sample size with an alpha level of 0.05 and a power of 0.8. The present study recruited more patients in comparison to the least necessary number, and the power of this study was 0.851 for detecting a significant difference between the two groups. Lastly, psychological factors are also important in rehabilitation following ACLR; however, the present study did not evaluate the psychological differences between the two groups. Further studies are needed to elucidate whether supervised rehabilitation can affect psychological states in the patients undergoing rehabilitation following ACLR. If so, it would be worth investigating whether that psychological improvement is associated with better functional outcomes.

## 5. Conclusions

Unlike previous studies that showed no significant difference between supervised and home-based rehabilitation, the current study suggests that supervision may lead to better outcomes in muscle strength, neuromuscular control, and patient-reported knee function up to 1 year after ACLR. The supervised rehabilitation group showed higher muscle strength of hamstring and quadriceps in nonoperative knees at 6 months and higher hamstring strength in operative and nonoperative knees at 1 year. The hamstring strength at deep flexion was also higher in both operative and nonoperative knees at 6 months and 1 year in the supervised rehabilitation group. Furthermore, the supervised rehabilitation group had better neuromuscular control assessed by AT and OSI in 6 months and 1 year. Lastly, the supervised rehabilitation group showed greater Lysholm score than the home-based rehabilitation group.

Although patients undergoing home-based rehabilitation may achieve an acceptable level of knee function and stability over the year, given the potential deficits and increased risk of ACL injury in the contralateral limbs, sufficient education may be needed for the patients who do not choose to undergo supervised rehabilitation.

## Figures and Tables

**Table 1:** Demographic data of participants in the supervised and home-based rehabilitation groups.

Baseline Characteristics	Supervised Rehabilitation (*n* = 13)	Home-Based Rehabilitation (*n* = 13)	*p*-Value
Sex (male/female)	10/3	9/4	
Age (years) ^1^	27.1 ± 7.2	28.6 ± 8.3	0.834
Height (cm) ^1^	175 ± 6.1	177 ± 5.3	0.671
Weight (kg) ^1^	73.1 ± 6.3	69.4 ± 9.4	0.764
Body mass index (kg/m^2^) ^1^	27.5 ± 1.4	25.4 ± 2.1	0.811
Injured side (right/left)	9/4	10/3	

^1^ Values are expressed as mean ± standard deviation.

**Table 2:** Serial changes in muscle ratio and strength of quadriceps and hamstring of enrolled subjects in the supervised rehabilitation group.

Supervised Rehabilitation Group (*n* = 13)	Pre-op. Baseline (Time 0)	Post-op. 6 Months (Time 1)	Post-op. 1 Year (Time 2)	*p*-Value			Mean (95% CI)		
				Time 0 × 1	Time 0 × 2	Time 1 × 2	Time 0 × 1	Time 0 × 2	Time 1 × 2
H to Q ratio (non-operative)	50.2 ± 11.4	46.2 ± 5.6	48.6 ± 7.8	0.492			4.0 (−6.3 to 14.3)	1.6 (−9.3 to 12.7)	−2.3 (−9.3 to 4.7)
				0.902	1.0	1.0			
H to Q ratio (operative)	46.7 ± 18.9	54.8 ± 14.4	50.5 ± 10.5	0.312			−8.1 (−28.1 to 11.8)	−3.8 (−16.6 to 8.9)	4.3 (−5.9 to 14.6)
				0.832	1.0	0.784			
Q (non-operative)	222.3 ± 48.9	269.8 ± 60.5	259.7 ± 42.6	0.051			−47.4 (−107.4 to 12.5)	−37.3 (−80.7 to 6.0)	10.1 (−25.7 to 45.9)
				0.145	0.102	1.0			
Q (operative)	114.9 ± 68.2	178.5 ± 37.7	227.7 ± 41.8	0.000			−63.5 (−117.1 to −9.8)	−112.8 (−165.6 to −59.9)	−49.3 (−85.3 to −13.2)
				0.019	0.000	0.008			
H (non-operative)	108.8 ± 25.9	124.4 ± 30.9	128.9 ± 29.9	0.058			−15.6 (−37.3 to 6.0)	−20.1 (−44.2 to 3.9)	−4.5 (−15.6 to 6.6)
				0.204	0.116	0.846			
H (operative)	55.0 ± 37.9	95.2 ± 21.4	113.3 ± 21.8	0.000			−40.2 (−66.9 to −13.6)	−58.3 (−85.9 to −30.6)	−18.1 (−28.8 to −7.3)
				0.004	0.000	0.002			
H, 100° flexion (non-operative)	93.4 ± 24.7	103.1 ± 18.6	115.7 ± 24.7	0.010			−9.7 (−21.9 to 2.4)	−22.3 (−43.3 to −1.3)	−12.6 (−26.4 to 1.2)
				0.137	0.036	0.078			
H, 100° flexion (operative)	39.7 ± 25.9	84.4 ± 22.6	98.9 ± 20.1	0.000			−44.7 (−66.4 to −22.9)	−59.2 (−80.5 to −38.0)	−14.6 (−25.9 to −3.1)
				0.000	0.000	0.012			

H = hamstring; Q = quadriceps; CI = confidence interval. Time 0 × 1, *p*-value between time 0 and 1; Time 0 × 2, *p*-value between time 0 and 2; Time 1 × 2, *p*-value between time 1 and 2. Note: The values expressed as Mean ± standard deviation. Measurement unit of quadriceps and hamstring muscle strength was Nm kg^−1^ × 100.

**Table 3:** Serial changes in muscle ratio and strength of quadriceps and hamstring of enrolled subjects in the home-based rehabilitation group.

Home-Based Rehabilitation Group (*n* = 13)	Pre-op. Baseline (Time 0)	Post-op. 6 Months (Time 1)	Post-op. 1 Year (Time 2)	*p*-Value			Mean (95% CI)		
				Time 0 × 1	Time 0 × 2	Time 1 × 2	Time 0 × 1	Time 0 × 2	Time 1 × 2
H to Q ratio (non-operative)	48.1 ± 8.1	44.7 ± 8.3	45.9 ± 6.0	0.208			3.5 (−2.3 to 9.3)	2.2 (−2.6 to 7.0)	−1.2 (−6.6 to 4.1)
				0.365	0.661	1.0			
H to Q ratio (operative)	52.4 ± 20.6	68.8 ± 24.6	63.1 ± 31.7	0.224			−16.4( −43.2 to 10.4)	−10.7 (−41.7 to 20.3)	5.7 (−11.3 to 22.7)
				0.345	1.0	1.0			
Q (non-operative)	207.4 ± 56.9	225.5 ± 44.8	220.0 ± 72.5	0.306			−18.1 (−43.9 to 7.6)	−12.7 (−46.5 to 21.1)	5.4 (−32.6 to 43.5)
				0.222	0.953	1.0			
Q (operative)	106.6 ± 53.8	144.8 ± 64.8	179.9 ± 77.2	0.000			−38.2 (−77.5 to 1.0)	−73.3 (−117.0 to −29.6)	−35.1 (−67.1 to −3.0)
				0.057	0.002	0.031			
H (non-operative)	96.0 ± 22.4	100 ± 23.7	100 ± 22.8	0.720			−4.0 (−17.2 to 9.3)	−4.0 (−22.1 to 14.1)	0 (−17.8 to 17.8)
				1.0	1.0	1.0			
H (operative)	54.8 ± 26.3	89.7 ± 30.6	93.4 ± 23.7	0.000			−34.9 (−55.3 to −14.5)	−38.5 (−57.3 to −19.8)	−3.6 (−21.6 to 14.4)
				0.001	0.000	1.0			
H, 100° flexion (non-operative)	80.0 ± 20.1	67.1 ± 19.4	78.8 ± 17.7	0.058			12.9 (−5.2 to 30.9)	1.2( −12.6 to 14.9)	−11.7 (−23.7 to 0.4)
				0.214	1.0	0.059			
H, 100° flexion (operative)	29.6 ± 13.6	53.2 ± 27.4	70.4 ± 19.2	0.000			−23.6 (−42.8 to −4.4)	−40.8 (−57.3 to −24.3)	−17.2 (−33.5 to −0.9)
				0.015	0.000	0.038			

H = hamstring; Q = quadriceps; CI = confidence interval. Time 0 × 1, *p*-value between time 0 and 1; Time 0 × 2, *p*-value between time 0 and 2; Time 1 × 2, *p*-value between time 1 and 2. Note: The values expressed as Mean ± standard deviation. Measurement unit of quadriceps and hamstring muscle strength was Nm kg^−1^ × 100.

**Table 4:** Serial changes in muscle ratio and strength of quadriceps and hamstring of enrolled subjects in the home-based rehabilitation group.

Supervised Rehabilitation Group (*n* = 13)	Pre-op. Baseline (Time 0)	Post-op. 6 Months (Time 1)	Post-op. 1 Year (Time 2)	*p*-Value			Mean (95% CI)		
				Time 0 × 1	Time 0 × 2	Time 1 × 2	Time 0 × 1	Time 0 × 2	Time 1 × 2
Q-AT (non-operative)	48.5 ± 12	31.5 ± 6	26.2 ± 5	0.000			17 (5.4 to 28.4)	22.3( 11.3 to 33.3)	5.4 (−1.4 to 12.1)
				0.005	0.000	0.141			
Q-AT (operative)	47.7 ± 13	40.8 ± 11	33.1 ± 10	0.002			6.9 (−2.7 to 16.6)	14.6 (3.9 to 25.3)	7.7 (−0.2 to 15.5)
				0.207	0.008	0.054			
H-AT (non-operative)	55.4 ± 13	49.2 ± 7	30.8 ± 11	0.000			6.1 (−7.8 to 20.0)	24.6 (12.6 to 36.6)	18.5 (7.6 to 29.3)
				0.727	0.000	0.001			
H-AT (operative)	63.1 ± 11	50.8 ± 10	32.3 ± 9	0.000			12.3 (0.5 to 24.1)	30.8 (19.7 to 41.9)	18.5 (7.2 to 29.7)
				0.041	0.000	0.002			
OSI (non-operative)	1.4 ± 0.4	0.9 ± 0.3	0.6 ± 0.2	0.000			0.4 (0.2 to 0.6)	0.8 (0.5 to 1.0)	0.3 (0.2 to 0.4)
				0.000	0.000	0.000			
OSI (operative)	1.5 ± 0.6	1.0 ± 0.4	0.7 ± 0.2	0.000			0.5 (0.2 to 0.9)	0.8 (0.4 to 1.2)	0.2( 0.1 to 0.4)
				0.002	0.001	0.007			

H = hamstring; Q = quadriceps; AT = acceleration time; OSI = overall stability index; CI = confidence interval. Time 0 × 1, *p*-value between time 0 and 1; Time 0 × 2, *p*-value between time 0 and 2; Time 1 × 2, *p*-value between time 1 and 2. Note: The values expressed as mean ± standard deviation, measurement unit of acceleration time: millisecond (msec), measurement unit of postural stability: degree (°).

**Table 5:** Serial changes in acceleration time of quadriceps and hamstring, and dynamic postural stability of enrolled subjects in the home-based rehabilitation group.

Home-Based Rehabilitation Group (*n* = 13)	Pre-op. Baseline (Time 0)	Post-op. 6 Months (Time 1)	Post-op. 1 Year (Time 2)	*p*-Value			Mean (95% CI)		
				Time 0 × 1	Time 0 × 2	Time 1 × 2	Time 0 × 1	Time 0 × 2	Time 1 × 2
Q-AT (non-operative)	36.9 ± 17	30 ± 16	30.8 ± 16	0.355			6.9 (−6.6 to 20.4)	6.2 (−9.1 to 21.4)	−0.8 (−15 to 13.5)
				0.538	0.854	1.0			
Q-AT (operative)	45.4 ± 25	47.7 ± 37	27.7 ± 19	0.093			−2.3 (−31.7 to 27.1)	17.7 (−5.2 to 40.6)	20 (−6 to 46)
				1.0	0.160	0.160			
H-AT (non-operative)	60.8 ± 24	44.6 ± 11	43.8 ± 18	0.024			16.1 (−3.1 to 35.4)	16.9( −1.2 to 35.1)	0.8 (−9.9 to 11.4)
				0.727	0.000	0.001			
H-AT (operative)	66.9 ± 25	50 ± 24	47.7 ± 19	0.003			16.9 (0.5 to 33.4)	19.2 (6.1 to 32.4)	2.3 (−11.8 to 16.4)
				0.043	0.005	1.0			
OSI (non-operative)	2.3 ± 1.0	2.1 ± 0.6	1.9 ± 0.9	0.447			0.2 (−0.6 to 1.0)	0.4 (−0.4 to 1.1)	0.2 (−0.7 to 0.9)
				1.0	0.580	1.0			
OSI (operative)	1.8 ± 0.6	1.9 ± 0.6	2.0 ± 0.6	0.588			−0.1 (−0.7 to 0.5)	−0.2 (−0.7 to 0.3)	−0.1 (−0.5 to 0.3)
				1.0	0.982	1.0			

H = hamstring; Q = quadriceps; AT = acceleration time; OSI = overall stability index; CI = confidence interval. Time 0 × 1, *p* value between time 0 and 1; Time 0 × 2, *p* value between time 0 and 2; Time 1 × 2, *p* value between time 1 and 2. Note: The values expressed as mean ± standard deviation, measurement unit of acceleration time: millisecond (msec), measurement unit of postural stability: degree (°).

**Table 6:** Serial changes in Lysholm score of enrolled subjects in the supervised and home-based rehabilitation groups.

Lysholm Scores	Pre-op. Baseline (time 0)	Post-op. 6 Months (time 1)	Post-op. 1 Year (time 2)	*p*-Value			Mean (95% CI)		
				Time 0 × 1	Time 0 × 2	Time 1 × 2	Time 0 × 1	Time 0 × 2	Time 1 × 2
Supervised rehabilitation group	44.3 ± 8.7	71.3 ± 7.0	87.3 ± 5.8	0.000			−27 (−34.9 to −19)	−43 (−49.8 to −36.2)	−16 (−21 to 10.9)
				0.000	0.000	0.000			
Home-based rehabilitation group	46.3 ± 17.4	66.3 ± 16.8	75.6 ± 15.1	0.000			−20 (−32.6 to −7.6)	−29.3 (−38.9 to −19.9)	−9.3 (−18.3 to −0.3)
				0.002	0.000	0.042			

## Data Availability

The data presented in this study are available on request from the corresponding author. The data are not publicly available due to privacy reasons.
